# Information About Inequality of Opportunity Increases Downward Mobility Perceptions: A Population-Wide Randomized Survey Experiment

**DOI:** 10.3389/fpsyg.2022.868303

**Published:** 2022-05-04

**Authors:** Alexi Gugushvili

**Affiliations:** ^1^Department of Sociology and Human Geography, University of Oslo, Oslo, Norway; ^2^Nuffield College, University of Oxford, Oxford, United Kingdom; ^3^Institute of Philosophy and Sociology, Polish Academy of Sciences, Warsaw, Poland

**Keywords:** equality of opportunity, social mobility, perceived social mobility, randomized survey experiment, Georgia

## Abstract

Existing evidence which is primarily based on cross-sectional and observational data suggests that perceptions of doing worse or better than parents might be more important for various life outcomes than the conventional measures of mobility based on the objective indicators of socioeconomic position. In 2021, we commissioned a nationally representative survey in Georgia which included a population-wide randomized survey experiment. We confirmed the association between, on the one hand, perceived social mobility and, on the other hand, physical and mental health, satisfaction with life, and the perceived state of affairs in the country. More importantly, the experimental design allowed us to conclude that the perception of being downwardly mobile was causally determined by a short message shared with individuals that equality of opportunity in their country was low. Those who were given information that children’s socioeconomic position was strongly linked to their parents’ socioeconomic position were seven percentage points more likely than individuals in the control group to perceive themselves as being downwardly mobile. We extrapolate these findings to the broader context and argue that the messages about (in)equality of opportunity which individuals receive in their everyday lives might also shape their perceptions of social mobility in other countries.

## Introduction

Intergenerational social mobility is conventionally estimated using different measures of socioeconomic position (Simons et al., 2013). The choice of the measure matters as the extent and nature of social mobility could be different along different dimensions of socioeconomic standing (Torssander and Erikson, 2010; Bukodi and Goldthorpe, 2013; Campos-Matos and Kawachi, 2015). For instance, since the 1990s many, if not most, countries around the world have experienced an educational expansion (Marginson, 2016), which has affected the rates of intergenerational educational mobility (Bernardi et al., 2018). In turn, the evidence for a large number of European countries suggests that the levels of intergenerational downward mobility in occupational attainment have been increasing for those born after the 1960s (Goldthorpe, 2016; Bukodi et al., 2020). Further, moving up or down in the occupational hierarchy does not necessarily correspond to an intergenerational change in actual incomes associated with these positions (Zhou, 2019; Tomaskovic-Devey et al., 2020).

Even if individuals attain better occupational status and higher incomes, this does not automatically imply that they consider themselves as being better off than their parents. It is known that wellbeing is not only determined by individuals’ own experiences, but also by their perceptions of the state of society as a whole, i.e., so-called sociotropic evaluations of the environment in which individuals live (Sutherland and Morley, 2008; Hansford and Gomez, 2015). An emerging area of scholarship has begun to explore the trends, causes, and consequences of social mobility perceptions, but so far there are only a few sources of data on this aspect of social stratification research. In the United States, where appropriate data are available, about 20% of individuals born since the 1960s have reported being downwardly mobile when asked to compare their own standard of living to their parents’ standard of living (Berger and Engzell, 2020). The latter is puzzling considering that time-adjusted *per capita* national income in the United States in the same period more than tripled (World Bank, 2020).

Understanding the nature of perceived social mobility is a worthwhile research endeavor because perceptions might be more important for various attitudinal, behavioral, health, and wellbeing outcomes than individuals’ objective experiences (Lipset, 1992). Perception of social mobility has been recently identified as the predicter of various health and wellbeing outcomes (Sun et al., 2017). Three recent studies which explored the consequences of subjective social mobility found that perceived occupational mobility was linked with individuals’ life satisfaction in Germany (Präg and Gugushvili, 2021), in Russia a strong and consistent association was found between perceptions of social mobility and physical and mental health (Gugushvili and Präg, 2021), while in Poland perceived social mobility was a significant predictor of both self-reported physical health and psychological wellbeing (Gugushvili et al., 2022). If there is indeed a significant link between perception of social mobility and various individual-level outcomes, it is important to understand what factors explain why individuals think that they are doing worse or better in life in comparison with their parents.

There are only a handful of studies which investigate what determines individuals’ perceptions of social mobility (Kelley and Kelley, 2009; Berger and Engzell, 2020; Gugushvili, 2021b). The main finding from this research is that both individual and contextual explanations matter for perceptions of social mobility. From individual-level characteristics, in addition to objective mobility experiences, gender, marital status, and subjective socioeconomic position were found to be associated with perceptions of social mobility. However, all of these studies used cross-sectional and observational data which make it difficult to identify factors that causally affect perceptions of social mobility. For the macro-contextual environment, one of the main findings from this emerging scholarship is that the difference in economic development between individuals’ birth years and the year of interview is an important predictor of perceived social mobility (Kelley and Kelley, 2009; Gugushvili, 2021b). Therefore, exploring the determinants of perceived social mobility could be particularly relevant in countries which have experienced major economic transformation in recent decades.

The fundamental economic reforms in post-communist societies in Central and Eastern Europe could have affected individuals’ perceptions of social mobility as a significant share of populations in these countries became winners or losers in the post-communist transition (Tucker et al., 2002). Existing comparative research suggests that the effects of social mobility on health and wellbeing in post-communist countries are indeed more salient than in Western European societies (Präg and Gugushvili, 2020; Kaiser and Trinh, 2021). The former countries have also experienced radical transformation of their political regimes, some becoming full-fledged democracies while others turned into oppressive authoritarian states (Gugushvili, 2020). Recent research shows that in less democratic post-communist countries citizens acquire less information about inequality through state-controlled media channels and this practice has implications for population health (Gugushvili and Reeves, 2021). In turn, existing evidence from randomized survey experiments from different country contexts indicates that information given to individuals about the levels of income inequality or their position in the socioeconomic hierarchy causally affects their redistribution preferences, beliefs on the determinants of success in life, perceptions of the gap between the rich and the poor, and willingness to defend the existing socioeconomic system (Day and Fiske, 2017; Alesina et al., 2018; Hoy and Mager, 2021; Mijs and Hoy, 2021).

In the present study, using a population-wide randomized survey experiment conducted in January 2021 in Georgia, one of the post-communist countries in Eastern Europe, we investigate how information about intergenerational (in)equality of opportunity affects individuals’ own perceptions of being socially mobile. To our knowledge, this research question has not been previously explored in any other country. Therefore, we do not have specifically formulated hypotheses in terms of how sharing information about (in)equality of opportunity with individuals would affect their perceptions of being socially mobile. A straightforward expectation would be that, on the one hand, if individuals learn about a high level of equality of opportunity (i.e., socioeconomic advantages and disadvantages are not transmitted from one generation to another), they are more likely to perceive themselves as being socially mobile, while on the other hand, when individuals are informed that the level of social mobility is low, they are more likely to perceive themselves as being socially immobile.

## Materials and Methods

### Dataset

Data used in this study stem from a nationally representative survey conducted in Georgia, a post-communist country in Eastern Europe. Data were collected by a specialized survey organization, Caucasus Research Resource Center, on January 21–28, 2021 (the highest number of interviews, 19.7%, was conducted on 24th of January). The survey’s coverage was the country’s adult population and the sample size consisted of 1,270 completed interviews according to the American Association for Public Opinion Research’s (AAPOR) standard definitions which implies that at least 50% of survey questions were answered by the respondents. The survey’s minimum response rate (AAPOR RR1) was 29% calculated by the number of complete interviews divided by the number of interviews (complete and partial) plus the number of non-interviews (refusal, break-off, non-contacts, and others) plus all cases of unknown eligibility. The data collection mode was an interviewer-administered phone survey based on a sample design generated by the random digit dialing (RDD) survey approach. Primary sampling units consisted of individuals and each phone number was treated as a personal-use device. The sample strata were based on the capital city (Tbilisi), urban, and rural settlements. Out of all of the interviews, 10% were checked *via* call-backs and the average theoretical margin of error of the survey was 2.7%. Respondents gave free and informed consent to participate and the survey was conducted in adherence to all other AAPOR ethical research standards. The dataset is freely available *via* Open Science Framework platform (Gugushvili, 2021a).

### Sample Characteristics

The survey consisted of two sections. First, we collected data on the following social origin, sociodemographic, and socioeconomic characteristics of the respondents: age (mean 48.8, SD 16.2, min-max 18–90), gender (males 55.7%), parental education (mean 3.33, SD 1.49, min-max 1 = basic education, 5 = higher education), own education (mean 3.55, SD 1.39, min-max 1 = basic education, 5 = higher education), material deprivation (the cumulative index of involuntary unavailability of refrigerator, color TV, smartphone, tablet, car, air conditioner, washing machine, personal computer/laptop, hot water, and central heating—mean 3.87, SD 2.22, min-max 0–10), unemployment status (unemployed 20.6%), settlement type (capital city 36.4%), number of children (mean 0.92, SD 1.14, min-max 0–6), and being an internally displaced person (IDP status 7.2%). Many of these variables have been shown to be associated with social mobility perceptions (Kelley and Kelley, 2009; Gugushvili, 2021b; Gugushvili and Präg, 2021; Präg and Gugushvili, 2021). We also collected information on respondents’ physical health (“how would you assess your physical health?” and “good/very good” 49.8%), mental health (“how would you assess your mental health?” and “good/very good” 76.3%), satisfaction with life (those scoring eight or higher on a 0–10 scale after the question “how satisfied are you with your life as a whole today?” 25.0%), and respondents’ perception of the state of affairs in the country (those agreeing with the statement that “Georgia is moving in the right direction” 39.6%).

### Experiment

Between the first and second sections of the survey, respondents were randomly allocated to one of two treatment groups or a control group. By randomizing treatment, we could determine the effect of information given about existing levels of equality of opportunity in a society at large on the perception of being socially mobile or immobile by comparing differences in participants’ responses between the treatment and control groups. For those in the control group (33.5% of the sample), no information on social mobility was given; for those in T1 (33.1% of the sample), based on the adapted survey item from Alesina et al. (2018), the following information was shared as: “Studies suggest that social mobility in this country is high, which means that the chances of a poor kid staying poor as an adult are small. Many kids from poor families can become rich, which means that children’s socio-economic position when they grow up is not strongly related to their parents’ socio-economic position.” For those in T2 (33.5% of the sample), corresponding but opposite information was shared indicating that equality of opportunity in the country was low. All respondents were then asked what their socioeconomic position was in comparison with that of their parents at the same age with the answer options being “lower” (downwardly mobile = 26.8%), “about the same” (immobile = 47.3%), and “higher” (upwardly mobile = 25.9%). Since the sizes of our treatment and control groups were determined by the survey cost considerations, we could only refer to post-hoc power calculation which indicated that the study was well powered to identify treatment effects.

### Statistical Analysis

To estimate the treatment effect of information about (in)equality of opportunity on subjective social mobility perceptions in our randomized survey experiment, we compared differences in the average outcomes of interest between the treatment and control groups. For ease of interpretation, we fitted linear probability models after creating dummy variables for both treatment groups taking on the value 1 if the respondents belonged to a particular treatment group and 0 if the person belonged to the control group. Point estimates from linear probability regression model are very similar to the average marginal effects of the logistic model (Angrist and Pischke, 2009). The results in Supplementary Material, Supplementary Tables S1, S2, from logistic and multinomial (with perceived immobility as the base category) logistic regressions with corresponding odds ratios are essentially identical to the results reported in the main analysis. Our preferred linear probability model for treatment effects can be written in the following form:


$$Y_{j}=\beta_{0}+\beta_{1}T+X_{ϒ}+\varepsilon$$

where *β*_1_ shows the average difference in the share of respondents in the treatment and control groups that perceive themselves to be downwardly or upwardly mobile (i.e., the treatment effect); *X* is a vector of participants’ characteristics that account for a potential imbalance between the treatment and control groups in adjusted models; *β*_0_ is the intercept; and *ε* is the error term for the model. To analyze the heterogeneous treatment effects by participants’ social origin, sociodemographic, and socioeconomic characteristics, we interacted the treatment dummies with the characteristics collected prior to the experiment. In equation terms, we can write as:


$$Y_{j}=\beta_{0}+\beta_{1}T+X_{ϒ}+\beta_{3}T*X_{ϒ}+\varepsilon$$

where *β*_3_ captures the interaction effect of the treatment (T1 and T2) with individuals’ specific characteristics. In addition, to ensure the national representativeness of the results, the regression estimates were weighted using the 2014 National Census data for controlling respondents’ gender, age, ethnic identity, education, and residence. Population counts by groups were also calibrated using an iterative proportional fitting (raking) algorithm. All analyses were conducted in Stata/MP 17 (StataCorp, College Station, TX). A replication Stata do-file for the presented analysis is available *via* Open Science Framework (OSF) platform.

## Results

### Does Perceived Social Mobility Matter for Health and Wellbeing Outcomes?

**Figure 1:**
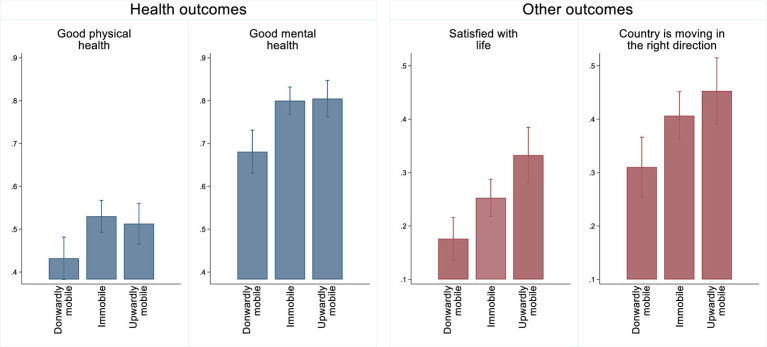
Perceived social mobility and various health and wellbeing outcomes, predictive margins from linear probability models. Bars show 95% confidence intervals. Models account for respondents’ age, gender, parental education, own education, material deprivation index, unemployment status, settlement type, number of children, IDP status, and the fixed effects for the interview date.

Before describing results from the population-wide survey experiment on the determinant of perceived social mobility, in Figure 1, we demonstrate how individuals’ perceptions of doing worse or better in life in comparison with their parents are linked to their health, life satisfaction, and the perception of the state of affairs in the country in which they live. Our linear probability models, with their binary outcome measures, also account for individuals’ social origin, and their sociodemographic and socioeconomic characteristics. Full results from these regressions are given in the Supplementary Material, Supplementary Table S3, while Figure 1 shows linear predictions from individuals’ perceptions of social mobility for good physical and mental health, satisfaction with life, and belief that the country is moving in the right direction. For both health outcome measures, those who perceive themselves to be downwardly mobile are also significantly less likely to report having good health. For instance, those who think they have done worse in life than their parents, have, respectively, 0.43 (CI95% 0.38, 0.48) and 0.68 (CI95% 0.63, 0.73) likelihood of reporting good physical and mental health, while corresponding estimates for individuals who perceive themselves to be immobile are 0.53 (CI95% 0.49, 0.57) and 0.80 (CI95% 0.76, 0.85). Further, those who perceive themselves to be upwardly mobile are also more satisfied with life (0.33 CI95% 0.28, 0.38) and more likely to think that the country is moving in the right direction (0.45 CI95% 0.39, 0.51).

### Does Information About (In)equality of Opportunity Affect Social Mobility Perceptions?

To understand if informing individuals about (in)equality of opportunity affects their perceptions of being socially mobile, we randomly divided the sample into two treatment groups and one control group. Those in treatment group 1 (T1) were told that equality of opportunity is high in Georgia and that the socioeconomic positions of individuals and their parents are not strongly related to each other, those in treatment group 2 (T2) were told that equality of opportunity is low, while individuals in the control group did not receive any information about (in)equality of opportunity. Table 1 presents descriptive statistics for an array of individual characteristics in the treatment and control groups. The mean values across groups are similar or very close to each other and all 95% confidence intervals for these estimates overlap. Bartlett’s equal-variances test also suggests that for all estimates, except that for being an internally displaced person (IDP), differences between means are not statistically significant. IDPs are one of the most disadvantaged groups in Georgia but their number in the sample is small (92 in total) which is the main reason why even minor absolute differences between groups are statistically significant in our equal-variances test (Shoemaker, 2003). For robustness of our estimates, we show the treatment effects both with and without adjusting for covariates.

**Table 1:** Descriptive statistics and the test of significant differences between treatment and control groups.

	Control group *N* = 425		T1: “Equality of opportunity is high” *N* = 420		T2: “Equality of opportunity is low” *N* = 425		Bartlett’s equal-variances test ( *p*-value)
	Mean	CI95	Mean	CI95	Mean	CI95	
Male	0.44	0.39–0.48	0.46	0.41–0.50	0.44	0.39–0.48	0.994
Age	48.84	47.30–50.38	49.42	47.81–51.02	48.29	46.79–49.79	0.445
Parental education	3.30	3.15–3.44	3.46	3.31–3.60	3.25	3.11–3.39	0.799
Respondents’ education	3.48	3.34–3.61	3.70	3.57–3.83	3.47	3.33–3.60	0.948
Material deprivation	3.94	3.73–4.15	3.73	3.52–3.93	3.96	3.74–4.18	0.559
Unemployment	0.20	0.16–0.24	0.21	0.17–0.25	0.20	0.17–0.24	0.868
Living in the capital	0.35	0.31–0.40	0.39	0.34–0.43	0.36	0.31–0.40	0.907
Number of children	0.92	0.82–1.03	0.88	0.77–0.98	0.95	0.84–1.07	0.156
IDP status	0.08	0.05–0.10	0.08	0.06–0.11	0.06	0.04–0.08	0.003
Date of interview	3.86	3.70–4.02	3.80	3.64–3.96	3.72	3.56–3.88	0.865

*The descriptive statistics for the entire sample is given in the methods’ section. For illustrative reasons, the first day of data collection is denoted as 1 and the last day as 8*.

**Figure 2:**
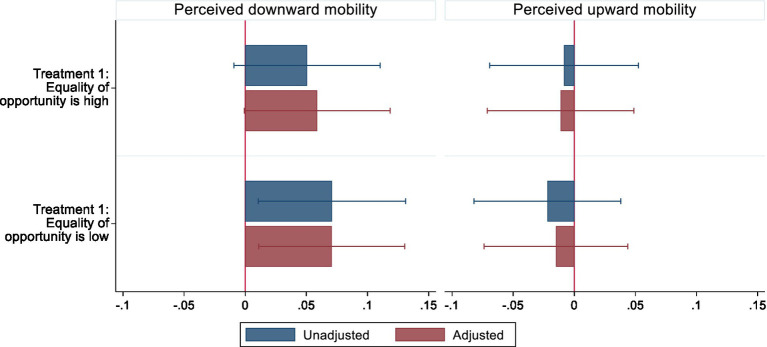
Effect of information about (in)equality of opportunity on the perception of being downwardly and upwardly mobile. Point estimates (with 95% confidence intervals) give the absolute difference between the treatment groups and control group in the proportion of respondents who perceived being downwardly or upwardly mobile when prompted that equality of opportunity is high (T1) or that equality of opportunity is low (T2). Adjusted linear probability models account for respondents’ age, gender, parental education, own education, material deprivation index, unemployment status, settlement type, number of children, IDP status, and the fixed effects for the interview date.

In Figure 2, we present the treatment effects expressed as the percentage point differences between the treated and the control groups for the perceptions of being downwardly and upwardly mobile. To reiterate, T1 implied informing individuals that equality of opportunity is high, while T2 implied telling individuals that equality of opportunity is low in the country. We observe that coefficients for T1 and T2 have positive signs for the perception of downward mobility and negative signs for the perception of upward mobility. Nonetheless, the point estimates for the perception of upward mobility are close to 0 and are not statistically significant. As for the perception of downward mobility, both treatments lead, at least, to a five percentage point higher likelihood of individuals’ declaring that they have done worse in life than their parents. For T1, in which individuals are informed equality of opportunity is high, the 95% confidence intervals overlap with 0 reference line and therefore, these effects are indistinguishable from mobility perceptions of individuals in the control group. In turn, T2, in which individuals are informed that in their country equality of opportunity is low, leads to a 7.1 (*β* 0.071, CI95 0.011, 0.130) percentage point higher likelihood of perceiving themselves to be downwardly mobile. These findings provide evidence that individuals’ perceptions of downward mobility are causally affected by information about inequality of opportunity. Full results for our treatment effects models are shown in the Supplementary Material, Supplementary Table S4.

### Are There Heterogeneous Treatment Effects?

**Figure 3:**
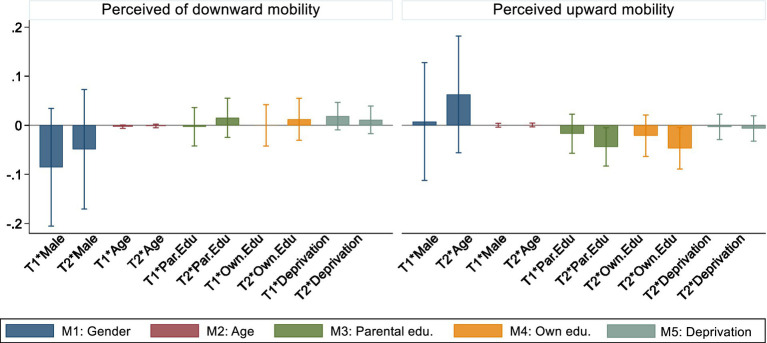
Interaction terms between treatment assignment (T1 and T2) and respondents’ characteristics collected before the experimental component of the survey. Interaction terms with 95% confidence intervals. Linear probability models account for the main effects of respondents’ age, gender, parental education, own education, and material deprivation index. In addition, individuals’ unemployment status, settlement type, number of children, IDP status, and the fixed effects for the interview date are also controlled for.

To understand if the effects of informing individuals about (in)equality of opportunity on their perceptions of social mobility vary depending on those individuals’ characteristics, we fit linear probability models with interaction terms between T1 and T2, on the one hand, and a number of social origin, sociodemographic, and socioeconomic variables, on the other hand. Figure 3 presents results for interactions between treatment groups and individuals’ gender, age, parental education, own education, and material deprivation. The full results of these models are shown in the Supplementary Material, Supplementary Tables S5, S6. For the perception of downward mobility, we observe that interaction terms are either indistinguishable from 0 or have large confidence intervals which overlap with 0. For the perception of upward mobility, on the other hand, we identify two significant effects. We find that educational attainment, both by respondents and their parents, decreases the likelihood that individuals who are informed about inequality of opportunity perceive themselves to be upwardly mobile. In the Supplementary Material, Supplementary Tables S7, S8, we also fit models with interaction terms between T1 and T2 and the remaining variables not shown in Figure 3. None of these interaction effects are statistically significant.

## Discussion

In recent decades, there has been increasing attention to the trends, causes, and consequences of subjective socioeconomic position across social science disciplines such as sociology, social psychology, and political science (Demakakos et al., 2008; Gidron and Hall, 2017; Zell et al., 2018). More recently scholars have started to analyze the determinants of perceived social mobility and its implications for various individual-level outcomes. The state of the art, however, relies on observational and cross-sectional data and corresponding statistical methods which do not allow the identification of causal factors determining social mobility perceptions. With our unique survey data collected in Georgia, we have been able to confirm the significant links between perceived social mobility and a number of outcomes such as good physical and mental health, satisfaction with life, and having a positive perception of the state of affairs in the country. However, these associations are likely to be affected by the unobserved heterogeneity of individuals. Some measures that are not included in the survey probably determine both individuals’ perceptions of being downwardly mobile, on the one hand, and having worse health and wellbeing outcomes, on the other hand.

Using the population-wide randomized experiment incorporated in the survey, we have been able to identify one of the causal factors affecting why some individuals perceive themselves to be downwardly mobile while other do not. A relatively short intervention over the phone lasting for about 15 seconds (the time required to read the statement in Georgian) and informing individuals about inequality of opportunity increased the chances of this treatment group of participants stating that they have experienced downward social mobility when compared with their parents by up to seven percentage points. Another intervention, informing individuals that the equality of opportunity is high, was also associated with a six percentage point greater perception of being downwardly mobile, but in this case, the large confidence intervals made the results indistinguishable from the control group. These findings contradict our initial expectation that information about low levels of equality of opportunity would lead to the lower likelihood of social mobility perceptions in both the downward and upward directions.

One of the explanations for why information about inequality of opportunity has an effect only on perceptions of downward mobility could be a well-known psychological concept of self-serving bias in causal attribution (Miller and Ross, 1975). Individuals are more likely to explain failure in life (i.e., downward mobility) by external factors (i.e., inequality of opportunity; Mezulis et al., 2004; Gugushvili, 2016). This mechanism works in the opposite direction for achieving success in life, which is more likely to be attributed by individuals to their own merits—talent, skill, and effort—regardless of the patterns of equality of opportunity in the country in which they live. Further, by analyzing the effects of interventions on specific groups of individuals with interactions terms between treatments and sociodemographic and socioeconomic characteristics, we also identified that those who are more educated are less likely to perceive themselves to be upwardly mobile if they are informed that equality of opportunity is low. Perhaps, more education allows individuals to see a stronger connection between inequality of opportunity and lower chances of experiencing upward social mobility (van Hugten and van Witteloostuijn, 2018).

Our study has its limitations. First, the nature of experimental intervention was relatively modest consisting of short pieces of verbal information shared over the phone with individuals which could have been difficult to comprehend for some participants. A treatment with visualization effects actually depicting a strong/weak association between parental and children’s socioeconomic position might be more appropriate in future studies. Second, the question on perceived social mobility followed immediately after the treatment condition and hence it is unknown how long the identified significant effect of T2 on the perception of downward mobility might last. Including experimental components in ongoing panel survey projects in different countries and repeatedly asking about perceived social mobility could potentially answer this question. Third, due to survey cost considerations, the sample sizes of the treatment and control groups were relatively small which restricted our ability to identify smaller treatment effects such as the effect of T1 on perceived downward mobility. Fourth, the survey was conducted at the end of the first major wave of the COVID-19 pandemic in Georgia and this could have influenced individuals’ answers on their wellbeing and social mobility perceptions. Fifth, Georgia is a country which, in addition to post-communist transition, has experienced civil and interstate wars and radical political instability in recent decades, which can also affect the generalizability of our findings.

Notwithstanding these limitations, we can extrapolate the findings from our randomized population-wide survey experiment to the broader context. If individuals receive messages about inequality of opportunity in their everyday lives, then they are likely to perceive themselves as being downwardly mobile. We know that individuals get information about inequality (and presumably about inequality of opportunity) *via* different channels such as following traditional and digital media, talking with friends and neighbors, and observing various forms of inequality at work, in schools, universities, government institutions, and other public spaces (Gugushvili et al., 2020; Gugushvili and Reeves, 2021). In turn, downward mobility, as we have shown in the current study, is associated with poor health, low wellbeing, and dissatisfaction with the state of affairs in the country. There might be a gap, however, between perceptions which individuals hold about equality of opportunity and the actual equality of opportunity (Kluegel and Smith, 1986; Alesina et al., 2018; Davidai, 2018; Cheng and Wen, 2019) and this gap can be determined by, among other factors, individuals’ political ideology and their social justice considerations (Alesina and La Ferrara, 2005; Osberg and Smeeding, 2006; Chambers et al., 2015; Day and Fiske, 2017). For instance, some studies show that in the United States, individuals hold more or less positive perceptions of equality of opportunity compared to the objective chances of experiencing social mobility and achieving the American Dream (Kraus, 2015; Kraus and Tan, 2015; Cheng and Wen, 2019).

Further, in contexts where inequality is increasing, individuals are more likely to think that socioeconomic outcomes are determined by factors that are beyond their control. Hence, if individuals underestimate objective inequality (Chambers et al., 2014; Davidai, 2018; Knell and Stix, 2020), this might reinforce their perceptions of social mobility. In future research, the experiment presented in this article on the effect of information about (in)equality of opportunity on the perception of being socially mobile can be replicated across country contexts or in various localities within countries that are characterized with different levels of inequality (Hauser and Norton, 2017; Solt, 2020). The latter is important because the perceptions of social mobility levels together with perceived own social mobility and future prospects of upward mobility can affect and motivate individuals’ persistence in academic and occupational realms, their health and wellbeing outcomes, and dissatisfaction with the state of affairs in the country (Ritterman Weintraub et al., 2015; Browman et al., 2017; Gugushvili and Kaiser, 2020; Heiserman et al., 2020).

## Data Availability Statement

The dataset used in this study is freely available *via* Open Science Framework platform: https://osf.io/7n2xf/.

## Ethics Statement

Ethical review and approval was not required for the study on human participants in accordance with the local legislation and institutional requirements. Written informed consent for participation was not required for this study in accordance with the national legislation and the institutional requirements.

## Author Contributions

The author confirms being the sole contributor of this work and has approved it for publication.

## Funding

This work was supported by the University of Oslo Research Support Unit and the Polish National Science Centre grant (Program SONATA14), grant number UMO-2018/31/D/HS6/01877.

## Conflict of Interest

The author declares that the research was conducted in the absence of any commercial or financial relationships that could be construed as a potential conflict of interest.

## Publisher’s Note

All claims expressed in this article are solely those of the authors and do not necessarily represent those of their affiliated organizations, or those of the publisher, the editors and the reviewers. Any product that may be evaluated in this article, or claim that may be made by its manufacturer, is not guaranteed or endorsed by the publisher.
