# Supplementary material for: Information About Inequality of Opportunity Increases Downward Mobility Perceptions: A Population-Wide Randomized Survey Experiment
Source: Front Psychol. 2022 May 4;13:868303. doi: 10.3389/fpsyg.2022.868303 (PMC9115553; doi:10.3389/fpsyg.2022.868303)
Supplement: Supplementary file 1 [file Table_1.DOCX]

Supplementary Material

Table S1. Effect of information about (in)equality of opportunity on the perception of being downwardly and upwardly mobile, odds ratios from logistic models.

|  | *Model 1: Perceived downward mobility* | *Model 2: Perceived downward mobility* | *Model 3: Perceived upward mobility* | *Model 4: Perceived upward mobility* |
| --- | --- | --- | --- | --- |
| Treatment condition |  |  |  |  |
| T1: Equality of opportunity is high | 1.308 | 1.391 | 0.958 | 0.930 |
|  | [0.952,1.797] | [0.999,1.937] | [0.701,1.308] | [0.671,1.289] |
| Control group | Reference | Reference | Reference | Reference |
| T2: Equality of opportunity is low | 1.444* | 1.480* | 0.891 | 0.921 |
|  | [1.055,1.977] | [1.068,2.050] | [0.651,1.220] | [0.667,1.273] |
| Age | –––––––––– | 0.997 | –––––––––– | 1.014** |
|  |  | [0.988,1.006] |  | [1.004,1.024] |
| Male | –––––––––– | 1.066 | –––––––––– | 0.878 |
|  |  | [0.811,1.401] |  | [0.665,1.159] |
| Parental education | –––––––––– | 1.141* | –––––––––– | 0.801*** |
|  |  | [1.020,1.276] |  | [0.712,0.900] |
| Respondents’ education | –––––––––– | 0.838** | –––––––––– | 1.224** |
|  |  | [0.745,0.942] |  | [1.083,1.384] |
| Material deprivation | –––––––––– | 1.211*** | –––––––––– | 0.823*** |
|  |  | [1.125,1.302] |  | [0.764,0.886] |
| Unemployment | –––––––––– | 1.001 | –––––––––– | 1.014 |
|  |  | [0.711,1.410] |  | [0.712,1.445] |
| Living in the capital | –––––––––– | 1.156 | –––––––––– | 0.706* |
|  |  | [0.864,1.546] |  | [0.523,0.953] |
| Number of children | –––––––––– | 1.015 | –––––––––– | 0.911 |
|  |  | [0.900,1.145] |  | [0.800,1.039] |
| IDP status | –––––––––– | 1.920** | –––––––––– | 1.330 |
|  |  | [1.239,2.975] |  | [0.823,2.150] |
| Interview date |  |  |  |  |
| 21 January | –––––––––– | Reference | –––––––––– | Reference |
| 22 January | –––––––––– | 0.837 | –––––––––– | 0.981 |
|  |  | [0.504,1.390] |  | [0.576,1.671] |
| 23 January | –––––––––– | 0.988 | –––––––––– | 1.025 |
|  |  | [0.590,1.657] |  | [0.602,1.743] |
| 24 January | –––––––––– | 0.806 | –––––––––– | 0.993 |
|  |  | [0.491,1.324] |  | [0.594,1.662] |
| 25 January | –––––––––– | 0.747 | –––––––––– | 1.171 |
|  |  | [0.444,1.255] |  | [0.703,1.951] |
| 26 January | –––––––––– | 0.753 | –––––––––– | 1.039 |
|  |  | [0.453,1.253] |  | [0.614,1.760] |
| 27 January | –––––––––– | 0.675 | –––––––––– | 2.583 |
|  |  | [0.198,2.300] |  | [0.785,8.496] |
| Model statistics |  |  |  |  |
| AIC | 1419.346 | 1370.232 | 1401.815 | 1342.239 |
| BIC | 1434.669 | 1461.943 | 1417.137 | 1433.950 |
| Observations | 1,208 | 1,208 | 1,208 | 1,208 |
| Pseudo R^2^ | 0.0631 | 0.0508 | 0.0004 | 0.0527 |

*Notes:* * p<0.05, ** p<0.01, *** p<0.001. 95% confidence intervals in brackets.

Table S2. Effect of information about (in)equality of opportunity on the perception of being downwardly and upwardly mobile, odds ratios from multinomial logistic models.

|  | *Model 1 (ref. perceived immobility)* | | *Model 2 (ref. perceived immobility)* | |
| --- | --- | --- | --- | --- |
|  | *Perceived downward mobility* | *Perceived upward mobility* | *Perceived downward mobility* | *Perceived upward mobility* |
| Treatment condition |  |  |  |  |
| T1: Equality of opportunity is high | 1.334 | 1.057 | 1.415 | 1.040 |
|  | [0.950,1.872] | [0.758,1.475] | [0.998,2.007] | [0.737,1.467] |
| Control group | Reference | Reference | Reference | Reference |
| T2: Equality of opportunity is low | 1.453* | 1.017 | 1.507* | 1.056 |
|  | [1.039,2.031] | [0.727,1.422] | [1.068,2.126] | [0.749,1.487] |
| Age | –––––––––– | –––––––––– | 1.001 | 1.014** |
|  |  |  | [0.992,1.011] | [1.003,1.025] |
| Male | –––––––––– | –––––––––– | 1.037 | 0.888 |
|  |  |  | [0.776,1.386] | [0.661,1.193] |
| Parental education | –––––––––– | –––––––––– | 1.068 | 0.819** |
|  |  |  | [0.950,1.201] | [0.725,0.926] |
| Respondents’ education | –––––––––– | –––––––––– | 0.882* | 1.172* |
|  |  |  | [0.779,0.997] | [1.031,1.332] |
| Material deprivation | –––––––––– | –––––––––– | 1.155*** | 0.866*** |
|  |  |  | [1.070,1.248] | [0.801,0.936] |
| Unemployment | –––––––––– | –––––––––– | 1.003 | 1.014 |
|  |  |  | [0.698,1.441] | [0.696,1.477] |
| Living in the capital | –––––––––– | –––––––––– | 1.046 | 0.717* |
|  |  |  | [0.769,1.424] | [0.522,0.985] |
| Number of children | –––––––––– | –––––––––– | 0.982 | 0.906 |
|  |  |  | [0.865,1.114] | [0.790,1.040] |
| IDP status | –––––––––– | –––––––––– | 2.503*** | 1.974* |
|  |  |  | [1.497,4.184] | [1.123,3.468] |
| Interview date |  |  |  |  |
| 21 January | –––––––––– | –––––––––– | Reference | Reference |
| 22 January | –––––––––– |  | 0.815 | 0.912 |
|  |  | –––––––––– | [0.471,1.408] | [0.514,1.619] |
| 23 January | –––––––––– |  | 0.990 | 1.019 |
|  |  | –––––––––– | [0.568,1.724] | [0.575,1.803] |
| 24 January | –––––––––– |  | 0.778 | 0.903 |
|  |  | –––––––––– | [0.458,1.324] | [0.520,1.568] |
| 25 January | –––––––––– |  | 0.761 | 1.064 |
|  |  | –––––––––– | [0.437,1.327] | [0.616,1.836] |
| 26 January | –––––––––– |  | 0.731 | 0.930 |
|  |  | –––––––––– | [0.425,1.259] | [0.530,1.632] |
| 27 January | –––––––––– |  | 1.023 | 2.608 |
|  |  | –––––––––– | [0.240,4.369] | [0.640,10.626] |
| Model statistics |  |  |  |  |
| AIC | 2586.660 |  | 2503.654 |  |
| BIC | 2617.305 |  | 2697.330 |  |
| Observations | 1,208 |  | 1,208 |  |
| Pseudo R^2^ | 0.0022 |  | 0.0503 |  |

*Notes:* * p<0.05, ** p<0.01, *** p<0.001. 95% confidence intervals in brackets.

**Table S3.** Perceived social mobility and various health and wellbeing outcomes, point estimates from linear probability models.

|  | *Model 1: Good physical health* | *Model 2: Good mental health* | *Model 3: Satisfied with life* | *Model 4: County is moving in the right direction* |
| --- | --- | --- | --- | --- |
| Perceived social mobility |  |  |  |  |
| Downward mobility | -0.098** | -0.119*** | -0.077** | -0.096** |
|  | [-0.160,-0.036] | [-0.179,-0.059] | [-0.129,-0.024] | [-0.168,-0.024] |
| Immobile | Reference | Reference | Reference | Reference |
| Upward mobility | -0.017 | 0.005 | 0.080* | 0.046 |
|  | [-0.078,0.044] | [-0.049,0.058] | [0.017,0.143] | [-0.031,0.123] |
| Age | -0.012*** | -0.004*** | -0.004*** | 0.001 |
|  | [-0.013,-0.010] | [-0.006,-0.003] | [-0.005,-0.002] | [-0.001,0.003] |
| Male | 0.144*** | 0.054* | -0.000 | 0.008 |
|  | [0.092,0.196] | [0.008,0.101] | [0.051,0.050] | [0.055,0.071] |
| Parental education | -0.005 | 0.012 | 0.005 | -0.004 |
|  | [0.027,0.016] | [0.007,0.032] | [0.015,0.025] | [0.030,0.021] |
| Respondents’ education | 0.029** | 0.006 | 0.017 | 0.009 |
|  | [0.007,0.051] | [-0.014,0.026] | [-0.004,0.037] | [-0.018,0.036] |
| Material deprivation | -0.031*** | -0.023*** | -0.033*** | -0.012 |
|  | [-0.044,-0.017] | [-0.035,-0.010] | [-0.046,-0.020] | [-0.028,0.004] |
| Unemployment | -0.103** | -0.077* | -0.107*** | -0.155*** |
|  | [-0.169,-0.037] | [-0.139,-0.016] | [-0.167,-0.048] | [-0.230,-0.081] |
| Living in the capital | 0.021 | -0.052* | -0.049 | -0.067* |
|  | [-0.035,0.077] | [-0.103,-0.001] | [-0.101,0.004] | [-0.133,-0.001] |
| Number of children | -0.004 | 0.002 | 0.005 | 0.008 |
|  | [-0.027,0.020] | [-0.019,0.024] | [-0.017,0.028] | [-0.020,0.035] |
| IDP status | -0.037 | 0.029 | -0.105** | -0.065 |
|  | [-0.134,0.059] | [-0.056,0.115] | [-0.179,-0.032] | [-0.181,0.051] |
| Interview date |  |  |  |  |
| 21 January | Reference | Reference | Reference | Reference |
| 22 January | 0.031 | 0.014 | -0.025 | -0.045 |
|  | [-0.074,0.135] | [-0.081,0.109] | [-0.118,0.068] | [-0.168,0.078] |
| 23 January | -0.031 | 0.055 | 0.03 | -0.076 |
|  | [-0.136,0.073] | [-0.036,0.146] | [-0.066,0.126] | [-0.197,0.045] |
| 24 January | -0.016 | 0.002 | -0.004 | -0.044 |
|  | [-0.117,0.086] | [-0.090,0.094] | [-0.096,0.088] | [-0.163,0.076] |
| 25 January | -0.009 | 0.016 | -0.027 | -0.087 |
|  | [-0.111,0.092] | [-0.076,0.108] | [-0.119,0.066] | [-0.207,0.033] |
| 26 January | -0.071 | -0.048 | 0.014 | -0.045 |
|  | [-0.172,0.031] | [-0.144,0.047] | [-0.078,0.106] | [-0.167,0.077] |
| 27 January | 0.119 | 0.021 | 0.07 | -0.284* |
|  | [-0.116,0.354] | [-0.205,0.247] | [-0.224,0.363] | [-0.543,-0.025] |
| 28 January | 0.028 | 0.240* | 0.201 | -0.024 |
|  | [-0.315,0.371] | [0.054,0.426] | [-0.339,0.741] | [-0.742,0.693] |
| Intercept | 1.097*** | 1.040*** | 0.527*** | 0.487*** |
|  | [0.928,1.267] | [0.881,1.199] | [0.364,0.690] | [0.283,0.691] |
| Model statistics |  |  |  |  |
| AIC | 1456.749 | 1250.435 | 1280.51 | 1366.997 |
| BIC | 1553.586 | 1347.273 | 1376.95 | 1459.899 |
| Observations | 1208 | 1208 | 1183 | 982 |
| Adjusted R^2^ | 0.231 | 0.087 | 0.100 | 0.032 |

*Notes:* * p<0.05, ** p<0.01, *** p<0.001. 95% confidence intervals in brackets.

Table S4. Effect of information about (in)equality of opportunity on the perception of being downwardly and upwardly mobile, point estimates from linear probability models.

|  | *Model 1: Perceived downward mobility* | *Model 2: Perceived downward mobility* | *Model 3: Perceived upward mobility* | *Model 4: Perceived upward mobility* |
| --- | --- | --- | --- | --- |
| Treatment condition |  |  |  |  |
| T1: Equality of opportunity is high | 0.051 | 0.059 | -0.008 | -0.011 |
|  | [-0.009,0.110] | [-0.001,0.119] | [-0.069,0.052] | [-0.071,0.049] |
| Control group | Reference | Reference | Reference | Reference |
| T2: Equality of opportunity is low | 0.071* | 0.071* | -0.022 | -0.015 |
|  | [0.011,0.131] | [0.011,0.130] | [-0.082,0.038] | [-0.074,0.044] |
| Age | –––––––––– | -0.001 | –––––––––– | 0.002** |
|  |  | [-0.002,0.001] |  | [0.001,0.004] |
| Male | –––––––––– | 0.014 | –––––––––– | -0.017 |
|  |  | [-0.036,0.065] |  | [-0.068,0.033] |
| Parental education | –––––––––– | 0.025* | –––––––––– | -0.039*** |
|  |  | [0.004,0.046] |  | [-0.060,-0.019] |
| Respondents’ education | –––––––––– | -0.033** | –––––––––– | 0.036** |
|  |  | [-0.055,-0.011] |  | [0.014,0.057] |
| Material deprivation | –––––––––– | 0.037*** | –––––––––– | -0.034*** |
|  |  | [0.023,0.050] |  | [-0.046,-0.021] |
| Unemployment | –––––––––– | -0.000 | –––––––––– | 0.002 |
|  |  | [-0.066,0.066] |  | [-0.059,0.063] |
| Living in the capital | –––––––––– | 0.028 | –––––––––– | -0.059* |
|  |  | [-0.026,0.082] |  | [-0.112,-0.006] |
| Number of children | –––––––––– | 0.003 | –––––––––– | -0.017 |
|  |  | [-0.019,0.025] |  | [-0.040,0.006] |
| IDP status | –––––––––– | 0.134** | –––––––––– | 0.053 |
|  |  | [0.035,0.233] |  | [-0.041,0.147] |
| Interview date |  |  |  |  |
| 21 January | –––––––––– | Reference | –––––––––– | Reference |
| 22 January | –––––––––– | -0.034 | –––––––––– | -0.002 |
|  |  | [-0.134,0.066] |  | [-0.100,0.095] |
| 23 January | –––––––––– | -0.003 | –––––––––– | 0.003 |
|  |  | [-0.106,0.100] |  | [-0.095,0.101] |
| 24 January | –––––––––– | -0.043 | –––––––––– | -0.002 |
|  |  | [-0.141,0.054] |  | [-0.096,0.092] |
| 25 January | –––––––––– | -0.055 | –––––––––– | 0.029 |
|  |  | [-0.153,0.044] |  | [-0.068,0.126] |
| 26 January | –––––––––– | -0.054 | –––––––––– | 0.005 |
|  |  | [-0.153,0.044] |  | [-0.092,0.102] |
| 27 January | –––––––––– | -0.068 | –––––––––– | 0.199 |
|  |  | [-0.280,0.144] |  | [-0.063,0.461] |
| 28 January | –––––––––– | -0.319*** | –––––––––– | 0.762*** |
|  |  | [-0.507,-0.131] |  | [0.665,0.858] |
| Intercept | 0.227*** | 0.154 | 0.269*** | 0.321*** |
|  | [0.187,0.268] | [-0.014,0.322] | [0.226,0.312] | [0.153,0.489] |
| Model statistics |  |  |  |  |
| AIC | 1476.244 | 1427.929 | 1454.439 | 1397.387 |
| BIC | 1491.566 | 1524.766 | 1469.761 | 1494.225 |
| Observations | 1221 | 1208 | 1221 | 1208 |
| Adjusted R^2^ | 0.003 | 0.045 | -0.001 | 0.048 |

*Notes:* * p<0.05, ** p<0.01, *** p<0.001. 95% confidence intervals in brackets.

**Table S5.** Estimates for perceived downward mobility with interactions between treatment assignment (T1 and T2) and respondents’ characteristics collected before experiment, point estimates from linear probability models.

|  | *Model 1: Gender* | *Model 2:*  *Age* | *Model 3: Parental education* | *Model 4: Own education* | *Model 4: Material deprivation* |
| --- | --- | --- | --- | --- | --- |
| Treatment condition |  |  |  |  |  |
| T1: Equality of opportunity is high | 0.097* | 0.198* | 0.071 | 0.061 | -0.012 |
|  | [0.016,0.177] | [0.020,0.377] | [-0.079,0.221] | [-0.111,0.233] | [-0.128,0.103] |
| Control group | Reference | Reference | Reference | Reference | Reference |
| T2: Equality of opportunity is low | 0.092* | 0.144 | 0.021 | 0.028 | 0.027 |
|  | [0.014,0.170] | [-0.041,0.328] | [-0.125,0.167] | [-0.139,0.195] | [-0.091,0.146] |
| Interacting variable | 0.059 | 0.001 | 0.021 | -0.037* | 0.027* |
|  | [-0.024,0.142] | [-0.002,0.003] | [-0.009,0.051] | [-0.068,-0.005] | [0.005,0.048] |
| Interaction terms |  |  |  |  |  |
| Equality of opportunity is high | -0.086 | -0.003 | -0.003 | -0.000 | 0.019 |
|  | [-0.206,0.034] | [-0.006,0.001] | [-0.042,0.036] | [-0.042,0.042] | [-0.009,0.047] |
| Equality of opportunity is low | -0.049 | -0.001 | 0.015 | 0.012 | 0.011 |
|  | [-0.171,0.073] | [-0.005,0.002] | [-0.025,0.055] | [-0.031,0.055] | [-0.017,0.039] |
| Intercept | 0.135 | 0.084 | 0.168 | 0.169 | 0.191* |
|  | [-0.034,0.305] | [-0.105,0.273] | [-0.009,0.345] | [-0.017,0.356] | [0.009,0.373] |
| Model statistics |  |  |  |  |  |
| AIC | 1429.972 | 1429.617 | 1431.044 | 1431.51 | 1430.103 |
| BIC | 1537.003 | 1536.648 | 1538.076 | 1538.541 | 1537.135 |
| Observations | 1208 | 1208 | 1208 | 1208 | 1208 |
| Adjusted R^2^ | 0.045 | 0.045 | 0.044 | 0.044 | 0.045 |

*Notes:* * p<0.05, ** p<0.01, *** p<0.001. 95% confidence intervals in brackets. Linear probability models account for respondents’ age, gender, parental education, own education, material deprivation index, unemployment status, settlement type, number of children, IDP status, and the fixed effects for the interview date.

**Table S6.** Estimates for perceived upward mobility with interactions between treatment assignment (T1 and T2) and respondents’ characteristics collected before experiment, point estimates from linear probability models.

|  | *Model 1: Gender* | *Model 2:*  *Age* | *Model 3: Parental education* | *Model 4: Own education* | *Model 4: Material deprivation* |
| --- | --- | --- | --- | --- | --- |
| Treatment condition |  |  |  |  |  |
| T1: Equality of opportunity is high | -0.014 | -0.013 | 0.045 | 0.062 | 0.002 |
|  | [-0.097,0.069] | [-0.212,0.185] | [-0.102,0.192] | [-0.092,0.216] | [-0.121,0.125] |
| Control group | Reference | Reference | Reference | Reference | Reference |
| T2: Equality of opportunity is low | -0.042 | -0.041 | 0.130 | 0.149 | 0.011 |
|  | [-0.122,0.037] | [-0.236,0.154] | [-0.012,0.271] | [-0.004,0.302] | [-0.113,0.134] |
| Interacting variable | -0.041 | 0.002 | -0.020 | 0.058*** | -0.031** |
|  | [-0.126,0.044] | [-0.001,0.005] | [-0.050,0.010] | [0.026,0.091] | [-0.051,-0.010] |
| Interaction terms |  |  |  |  |  |
| Equality of opportunity is high | 0.008 | 0.000 | -0.017 | -0.021 | -0.003 |
|  | [-0.112,0.128] | [-0.004,0.004] | [-0.057,0.023] | [-0.064,0.021] | [-0.029,0.023] |
| Equality of opportunity is low | 0.063 | 0.001 | -0.044* | -0.047* | -0.007 |
|  | [-0.056,0.182] | [-0.003,0.004] | [-0.083,-0.005] | [-0.089,-0.005] | [-0.033,0.019] |
| Intercept | 0.332*** | 0.330** | 0.259** | 0.237* | 0.308*** |
|  | [0.160,0.504] | [0.128,0.531] | [0.076,0.442] | [0.049,0.426] | [0.127,0.489] |
| Model statistics |  |  |  |  |  |
| AIC | 1400.109 | 1401.289 | 1396.621 | 1396.606 | 1401.145 |
| BIC | 1507.14 | 1508.32 | 1503.653 | 1503.637 | 1508.176 |
| Observations | 1208 | 1208 | 1208 | 1208 | 1208 |
| Adjusted R^2^ | 0.047 | 0.046 | 0.05 | 0.05 | 0.047 |

*Notes:* * p<0.05, ** p<0.01, *** p<0.001. 95% confidence intervals in brackets. Linear probability models account for respondents’ age, gender, parental education, own education, material deprivation index, unemployment status, settlement type, number of children, IDP status, and the fixed effects for the interview date.

**Table S7.** Estimates for perceived downward mobility with interactions between treatment assignment (T1 and T2) and respondents’ characteristics collected before experiment, point estimates from linear probability models.

|  | *Model 1: Unemployed* | *Model 2:*  *Capital* | *Model 3: Children* | *Model 4:*  *IDP* |
| --- | --- | --- | --- | --- |
| Treatment condition |  |  |  |  |
| T1: Equality of opportunity is high | 0.065 | 0.095* | 0.021 | 0.045 |
|  | [-0.002,0.131] | [0.018,0.172] | [-0.055,0.097] | [-0.016,0.107] |
| Control group | Reference | Reference | Reference | Reference |
| T2: Equality of opportunity is low | 0.081* | 0.062 | 0.054 | 0.067* |
|  | [0.015,0.147] | [-0.011,0.135] | [-0.024,0.132] | [0.005,0.129] |
| Interacting variable | 0.027 | 0.053 | -0.017 | 0.062 |
|  | [-0.083,0.137] | [-0.035,0.141] | [-0.050,0.016] | [-0.092,0.217] |
| Interaction terms |  |  |  |  |
| Equality of opportunity is high | -0.029 | -0.096 | 0.042 | 0.161 |
|  | [-0.182,0.123] | [-0.217,0.024] | [-0.009,0.094] | [-0.067,0.389] |
| Equality of opportunity is low | -0.053 | 0.023 | 0.018 | 0.039 |
|  | [-0.209,0.103] | [-0.102,0.148] | [-0.032,0.069] | [-0.208,0.285] |
| Intercept | 0.146 | 0.134 | 0.169 | 0.161 |
|  | [-0.024,0.316] | [-0.035,0.304] | [-0.002,0.340] | [-0.008,0.330] |
| Model statistics |  |  |  |  |
| AIC | 1431.441 | 1427.9 | 1429.566 | 1429.596 |
| BIC | 1538.472 | 1534.931 | 1536.597 | 1536.627 |
| Observations | 1208 | 1208 | 1208 | 1208 |
| Adjusted R^2^ | 0.044 | 0.046 | 0.045 | 0.045 |

*Notes:* * p<0.05, ** p<0.01, *** p<0.001. 95% confidence intervals in brackets. Linear probability models account for respondents’ age, gender, parental education, own education, material deprivation index, unemployment status, settlement type, number of children, IDP status, and the fixed effects for the interview date.

**Table S8.** Estimates for perceived upward mobility with interactions between treatment assignment (T1 and T2) and respondents’ characteristics collected before experiment, point estimates from linear probability models.

|  | *Model 1: Gender* | *Model 2:*  *Age* | *Model 3: Parental education* | *Model 4: Respondents’ education* |
| --- | --- | --- | --- | --- |
| Treatment condition |  |  |  |  |
| T1: Equality of opportunity is high | -0.017 | -0.022 | 0.008 | 0.006 |
|  | [-0.085,0.052] | [-0.099,0.055] | [-0.072,0.089] | [-0.056,0.069] |
| Control group | Reference | Reference | Reference | Reference |
| T2: Equality of opportunity is low | -0.004 | -0.008 | -0.053 | -0.010 |
|  | [-0.070,0.063] | [-0.083,0.066] | [-0.130,0.024] | [-0.070,0.051] |
| Interacting variable | 0.013 | -0.062 | -0.025 | 0.149 |
|  | [-0.091,0.117] | [-0.151,0.026] | [-0.065,0.015] | [-0.014,0.311] |
| Interaction terms |  |  |  |  |
| Equality of opportunity is high | 0.023 | 0.028 | -0.022 | -0.209 |
|  | [-0.121,0.167] | [-0.095,0.151] | [-0.078,0.034] | [-0.424,0.006] |
| Equality of opportunity is low | -0.057 | -0.018 | 0.040 | -0.061 |
|  | [-0.199,0.085] | [-0.140,0.104] | [-0.013,0.094] | [-0.305,0.182] |
| Intercept | 0.311*** | 0.327*** | 0.338*** | 0.312*** |
|  | [0.142,0.481] | [0.154,0.499] | [0.166,0.510] | [0.143,0.480] |
| Model statistics |  |  |  |  |
| AIC | 1400.175 | 1400.831 | 1395.678 | 1397.479 |
| BIC | 1507.206 | 1507.862 | 1502.709 | 1504.51 |
| Observations | 1208 | 1208 | 1208 | 1208 |
| Adjusted R^2^ | 0.047 | 0.047 | 0.051 | 0.049 |

*Notes:* * p<0.05, ** p<0.01, *** p<0.001. 95% confidence intervals in brackets. Linear probability models account for respondents’ age, gender, parental education, own education, material deprivation index, unemployment status, settlement type, number of children, IDP status, and the fixed effects for the interview date.
